# Supplementary material for: Involvement of Vasopressin in Tissue Hypoperfusion during Cardiogenic Shock Complicating Acute Myocardial Infarction in Rats
Source: Int J Mol Sci. 2023 Jan 10;24(2):1325. doi: 10.3390/ijms24021325 (PMC9866678; doi:10.3390/ijms24021325)
Supplement: Supplementary file 1 [file ijms-24-01325-s001.zip › ijms-2143808-supplementary.pdf]

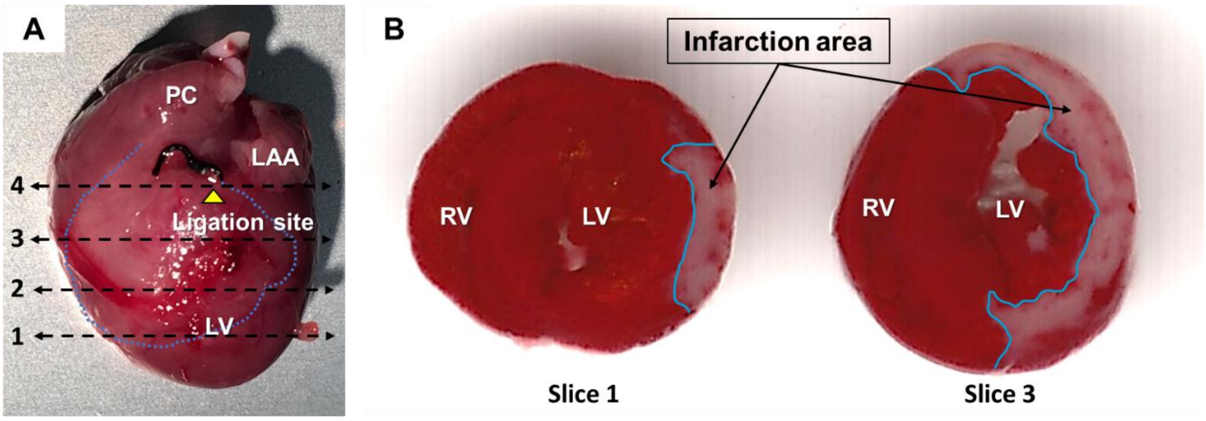

Supplemental Figure S1. Anatomical examination of the ischemic heart at D_1 after left coronary artery ligation. **A**: Picture showing the site of coronary ligation (yellow arrow) and the pale area of myocardial ischemia (blue dashed line). Dark dashed line with arrows represents the 4 cross-sections of the heart for histologic examination. **B**: Transversal cross-sections of the excised heart after incubation with 1% triphenyl-tetrazolium chloride. Myocardial infarction area, colored in white, is shown here by delimitation with the blue line in 2 slices. The infarcted area was determined by computerized planimetry with ImageJ® software in each slice to calculate the infarct area/total area ratio. PC: pulmonary conus, LAA: left atrial appendage, LV: left ventricle, RV: right ventricle.

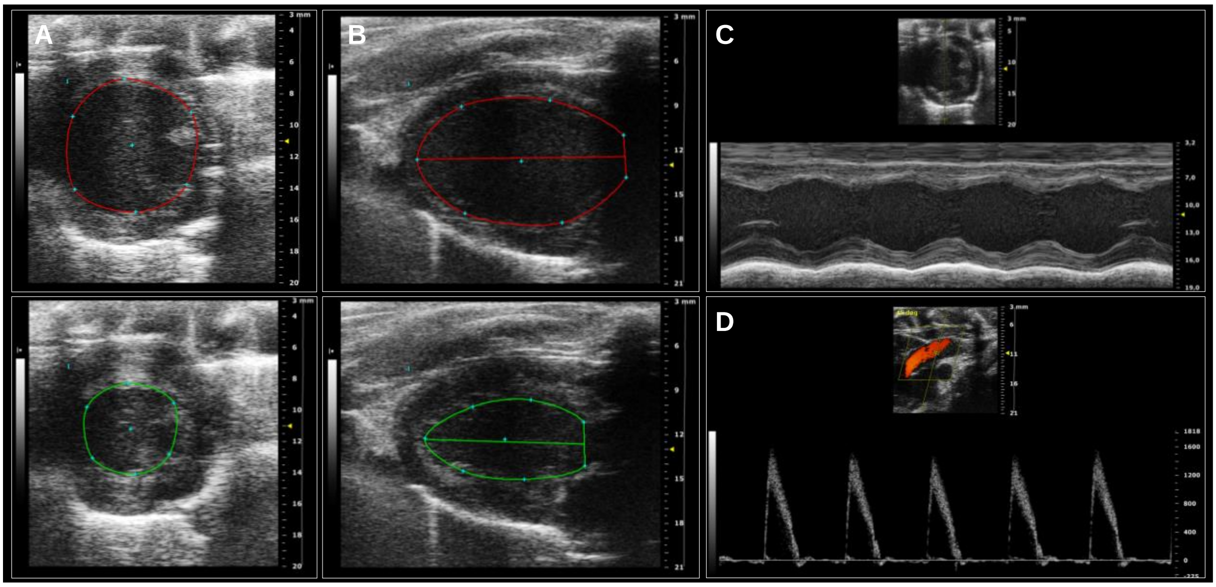

Supplemental Figure S2. Transthoracic echocardiographic recordings. Illustrations of (**A**) left ventricular endocardial end-diastolic (top panel) and end-systolic areas (bottom panel) in B-mode parasternal short axis view; (**B**) left ventricular endocardial end-diastolic (top panel) and end-systolic areas (bottom panel) in B-mode parasternal long axis view; (**C**) M-mode of parasternal short axis view at the level of papillary muscles and (**D**) pulsed wave Doppler of blood flow in the ascending aorta.

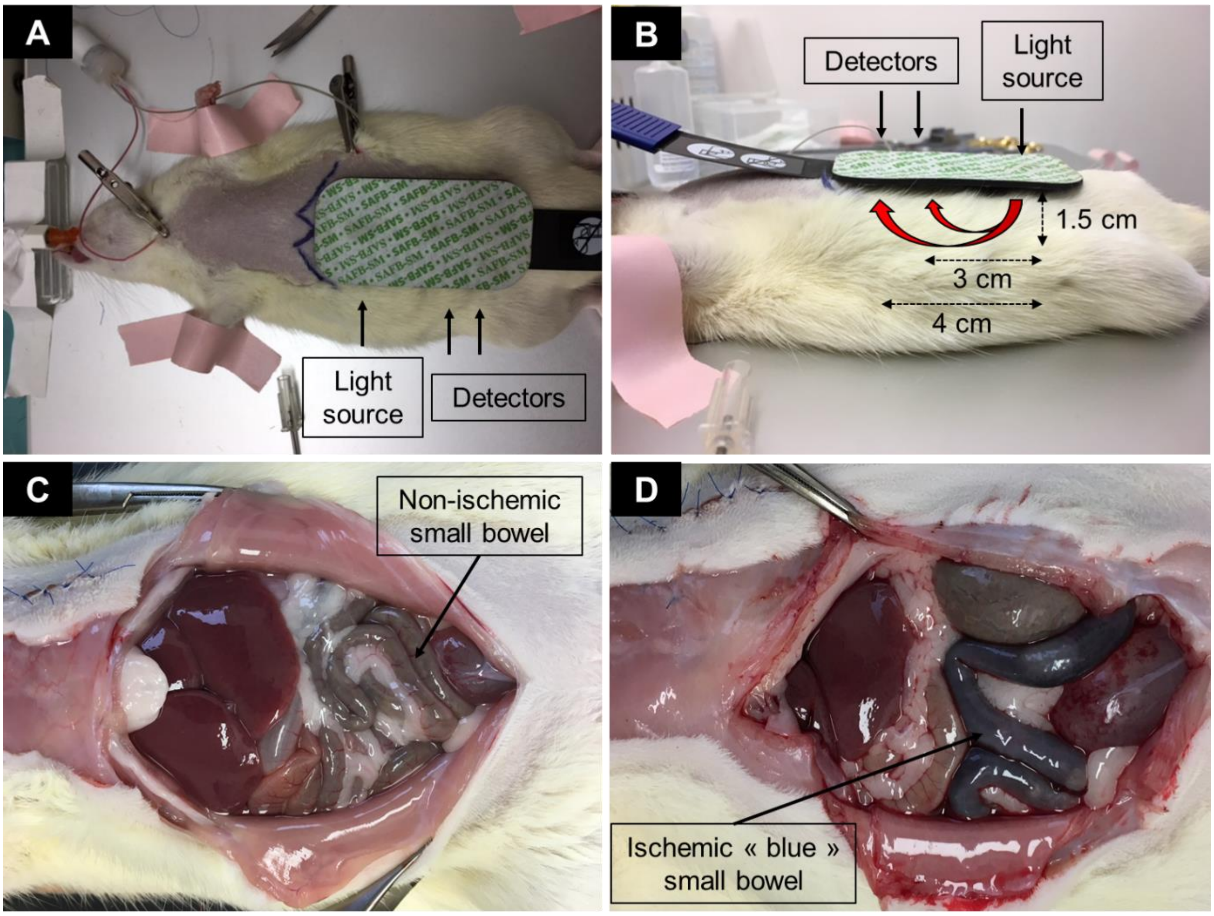

Supplemental Figure S3. Assessment of mesenteric oxygenation at D1 after left coronary artery ligation. **A-B**: Images showing the 2 positions of NIRS sensor, below anatomical delineation of the inferior edge of the sternum and ribs with light source located in the upper abdomen for the first measurement (A) and with the lower abdominal light source for the second measurement (B). **C-D**: Images showing laparotomy for intra-abdominal examination illustrating non-ischemic small bowel in a Sham (C) and small bowel ischemia in an animal with cardiogenic shock (D).
